# *Lyso-*phosphatidylethanolamine triggers immunity against necrotrophs by promoting JA-signaling and ROS-homeostasis

**DOI:** 10.1007/s11103-023-01385-x

**Published:** 2023-12-12

**Authors:** Ronny Vӧlz, Ki-Tae Kim, Mazen Alazem, William Harris, Sungkee Hwang, Yong-Hwan Lee

**Affiliations:** 1 Research Institute of Agriculture and Life Sciences, Seoul National University, Seoul, 08826 Korea; 2 Department of Agricultural Life Science, Sunchon National University, Suncheon, 57922 Korea; 3 Donald Danforth Plant Science Center, St Louis, Missouri USA; 4 Department of Agricultural Biotechnology, Seoul National University, Seoul, 08826 Korea; 5 NUTRA-PARK, Seoul, 06732 Korea; 6 Center for Fungal Genetic Resources, Seoul National University, Seoul, 08826 Korea; 7 Plant Immunity Research Center, Seoul National University, Seoul, 08826 Korea; 8 Center for Plant Microbiome Research, Seoul National University, Seoul, 08826 Korea

**Keywords:** Plant defense response, *Lyso*-phosphatidylethanolamine, Botrytis cinerea, Jasmonic acid signaling, Reactive oxygen species, FERONIA-COI1 mediated immune response, Phospho-lipid, *Arabidopsis thaliana*

## Abstract

Modulation of the plant defense response by bioactive molecules is of increasing interest. However, despite plant cell lipids being one of the major cellular components, their role in plant immunity remains elusive. We found that the exogenous application of the cell-membrane localized phospholipid *lyso*-phosphatidylethanolamine (LPE) reprograms the plant transcript profile in favor of defense-associated genes thereby priming the plant immune system. Exogenous LPE application to different Arabidopsis accessions increases resistance against the necrotrophic pathogens, *Botrytis cinerea* and *Cochliobolus heterostrophus*. We found that the immunity-promoting effect of LPE is repealed in the jasmonic acid (JA) receptor mutant *coi1*, but multiplied in the JA-hypersensitive mutant *feronia (fer-4).* The JA-signaling repressor JAZ1 is degraded following LPE administration, suggesting that JA-signaling is promoted by LPE. Following LPE-treatment, reactive oxygen species (ROS) accumulation is affected in *coi1* and *fer-4.* Moreover, FER signaling inhibitors of the RALF family are strongly expressed after LPE application, and RALF23 is internalized in stress granules, suggesting the LPE-mediated repression of FER-signaling by promoting RALF function. The *in-situ* increase of LPE-abundance in the LPE-catabolic mutants *lpeat1* and *lpeat2* elevates plant resistance to *B. cinerea*, in contrast to the endogenous LPE-deficient mutant *pla2-alpha*. We show that LPE increases plant resistance against necrotrophs by promoting JA-signaling and ROS-homeostasis, thereby paving the way for the LPE-targeted genomic engineering of crops to raise their ability to resist biotic threats.

**Supplementary Information:**

The online version contains supplementary material available at 10.1007/s11103-023-01385-x.

## Key message

Modulation of the plant defense response by bioactive molecules is of increasing interest. However, despite plant cell lipids being one of the major cellular components, their role in plant immunity remains elusive. We show that LPE increases plant resistance against necrotrophs by promoting JA-signaling and ROS-homeostasis, thereby paving the way for the LPE-targeted genomic engineering of crops to raise their ability to resist biotic threats.

**Supplementary Information:**

The online version contains supplementary material available at 10.1007/s11103-023-01385-x.

## Introduction

Plant development is highly plastic in its response to a changing environment. Plants can trigger specific differentiation programs, promote growth over differentiation, or favor defense strategies in response to biotic stresses. Although the molecular mechanisms by which plants integrate environmental and endogenous signals are not completely understood, there is a high degree of conservation between the various elements of plant signaling pathways (Marin-de la Rosa et al. 2015).

Plant-signaling lipids comprise a vast array of lipid classes including, fatty acid, phosphatidic acid, phospholipids, diacylglycerol, oxylipin, inositol phosphate, sphingolipid and N–acylethanolamine. Phospholipids participate in a wide spectrum of biological functions, including signal transduction, the storage of energy, and enabling the structural dynamics and integrity of cell membranes.

*Lyso*-phosphatidylethanolamine (LPE) is a minor component of the cell-membrane localized lipids and originates from the precursor phosphatidylethanolamine (PE). Phospholipase A2 hydrolyzes, the structural phospholipid PE, thereby generating LPE. Phosphoethanolamine is the precursor of PE and phosphatidylcholine (PC) and the rate-limiting enzyme PHOSPHORYLETHANOLAMINE CYTIDYLYLTRANSFERASE 1 (PECT1) modulates the PC: PE ratio in Arabidopsis (Canonne et al. 2011; Lee et al. 2005). The knockout of PECT1 impacts the PC: PE ratio to the disadvantage of PE (Mizoi et al. 2006). The two enzymes LYSOPHOSPHATIDYLETHANOLAMINE ACYLTRANSFERASE1 (LPEAT1) and LPEAT2 acetylate LPE with acyl-coenzyme A (Jasieniecka-Gazarkiewicz et al. 2017). The disruption of LPEAT1 and LPEAT2 increases the LPE content in leaves.

Lipid profiling of Arabidopsis plants revealed that PE contributes to around 6.45% and LPE approximately 0.026% of the total quantity of leaf lipids. LPE is a glycerolipid that is exogenously applied on a wide range of crops, e.g. green pepper, sweet cherry, strawberries, and tomatoes (Farag & Palta 1993; Amaro & Almeida 2013; Ozgen et al. 2015) to delay early senescence while simultaneously accelerating fruit ripening (Farag & Palta 1993; Ryu et al. 1997; Hong et al. 2009). Recently, we found that the application of exogenous LPE increases plant resistance against the hemibiotrophic pathogen *Pseudomonas syringae* (Volz et al. 2021).

Here, we show that the transcriptional profile of LPE-treated plants is enriched in immunity-associated transcripts. LPE modulates the ROS-homeostasis and JA-signaling thereby priming the plant for infection by necrotrophic invaders.

## Results

### LPE application reprograms defense-associated gene expression

**Fig. 1:**
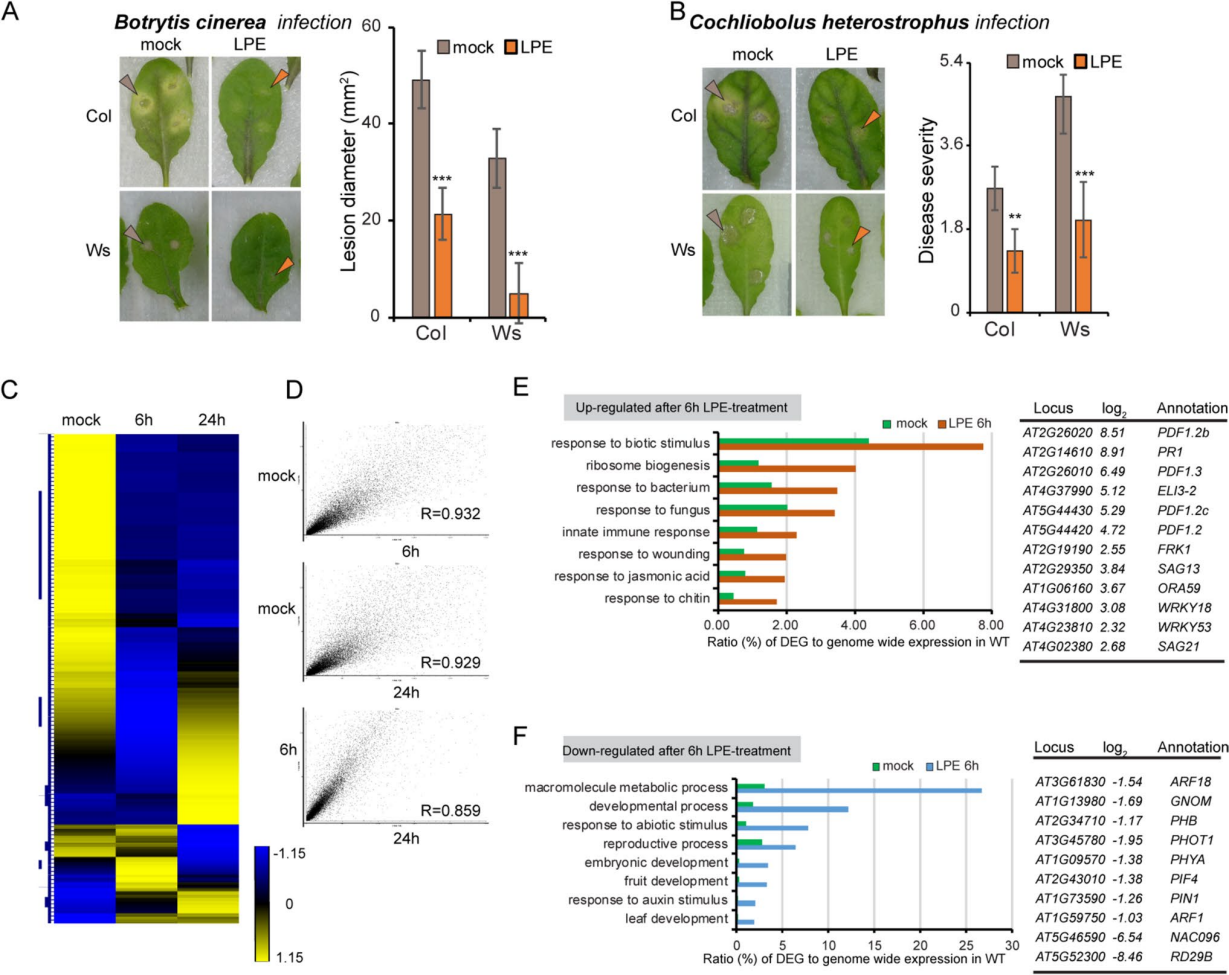
LPE application increases resistance against *Botrytis cinerea* and *Cochliobolus heterostrophus,* by promoting defense-associated transcriptional gene profiles. **A**, **B** LPE-immunity promoting effect was assessed by performing Infection experiments. Two *Arabidopsis thaliana* accessions *Columbia* (Col) and *Wasselevskaja* (Ws) were infected with *Botrytis cinerea* and *Cochliobolus heterostrophus* at 24 h after LPE and mock-pretreatment. The lesion formation and degree of infection were analyzed at 72 hpi. Error bars show ± SD. Three biological replicates were performed with similar results. Statistical significance was analyzed by one-way ANOVA. **C**, **D** Profiling of differentially-expressed gene (DEG) at 6 h and 24 h after LPE treatment. DEGs can be grouped in particular clusters demonstrating LPE-responding gene expression. **E** Upregulated gene expression at 6 h after LPE treatment contributes to defense and immunity-associated function. **F** Downregulated gene expression at 6 h after LPE treatment contributes to the growth and metabolic functions

While on the hunt for factors that activate the plant immune system, we identified a cell-membrane localized phospholipid LPE as a putative enhancer of defense-associated processes. We spray-applied LPE at 24 h before *Botrytis cinerea* inoculation and determined the lesion formation in two *Arabidopsis thaliana* accessions Columbia (Col) and *Wasselevskaja* (Ws). We found that the lesion formation at 72 h post inoculation (hpi) was significantly reduced in Col and Ws following LPE pretreatment (Fig. 1A). This result suggests that LPE pretreatment interferes with *B. cinerea*-triggered plant cell death and host-colonization. Furthermore, we analyzed whether LPE pretreatment also promotes resistance against the necrotrophic pathogen *Cochliobolus heterostrophus* C4 (Volz et al. 2020). We found that both Arabidopsis accessions exhibited diminished lesion formation at 72 hpi compared to the mock-treated control (Fig. 1B). These outcomes indicate a plant immunity-promoting effect of LPE pretreatment against necrotrophic pathogens.

To elucidate host-molecular processes that might be modulated by LPE treatment, we analyzed the transcript profile at 6 h, and 24 h after LPE application compared to the mock-treated control. When taking into account the expression profiles for all the detected transcripts a close to linear correlation coefficient (Pearson-correlation) was obtained with mock vs. 6 h (0.932), mock vs. 24 h (0.929), and 6 h vs 24 h (0.859) (Fig. 1C, D). The strict correlation suggests that LPE does not affect general gene expression in an unbiased way, but rather influences subsets of genes in particular biological processes. Hierarchical clustering of differentially expressed genes (DEGs) (*p* < 0.01), by the use of normalized FPKM values, revealed distinct changes in gene expression patterns in the course of LPE perception. We identified 1914 genes that were upregulated and 1,111 that were downregulated 6 h after LPE administration, while after 24 h, 2938 genes showed elevated transcript levels and 1659 genes exhibited reduced expression levels (Fig. S1A, Table S1). Upregulated genes at 6 h after LPE application were grouped into gene-ontology (GO) terms showing the upregulation of response to biotic stimulus, bacterium, fungus wounding, chitin, and jasmonic acid (Fig. 1E, S1D). Notably, marker genes for jasmonic acid signaling showed highly elevated transcript levels, e.g. *PDF1.2a/b/c, PDF1.3, ORA59*, but also genes involved in PTI response, salicylic acid and senescence were upregulated, such as *PR1, FRK1, WRKY53, SAG13,* and *SAG21*. By contrast, GO term analysis for downregulated genes at 6 h after LPE application highlighted gene functions in processes that contribute to growth, development, and metabolic processes (Fig. 1F).

This analysis suggests that LPE perception triggers regulatory pathways that favor defense-associated processes over plant growth. Furthermore, gene-regulatory networks are stimulated by LPE that contribute to defense against necrotrophic pathogens.

**Fig. 2:**
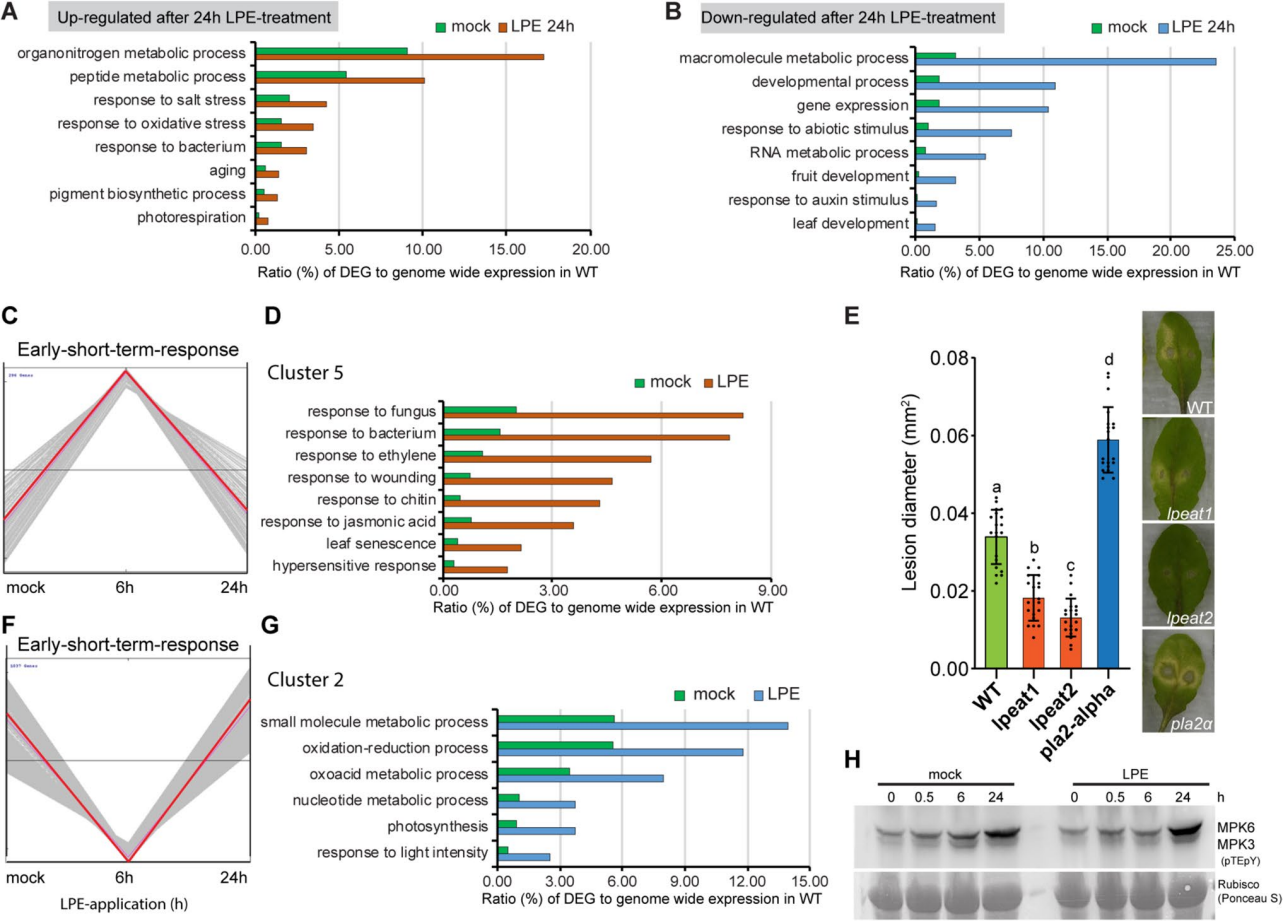
LPE-treatment changes defense and growth-related transcript profiles beneficial for immunity-associated signatures. **A** Upregulated gene expression at 24 h after LPE treatment contributes to defense and senescence-associated function. **B** Downregulated gene expression at 24 h after LPE treatment contributes to growth and metabolic functions. **C**, **D** Short-term response after LPE application promotes in particular defense-associated gene expression. Error bars show ± SD. Statistical significance was analyzed by one-way ANOVA. Also, see Fig. S1B. **E** Susceptibility assays of the LPE-accumulating biosynthesis mutants *lpeat1* and *lpeat2*, and the LPE-deficient mutant *pla2alpha* after *Botrytis cinerea* infection. The lesion formation and degree of infection were analyzed at 72 hpi. Error bars show ± SD. Three biological replicates were performed with similar results. Statistical significance was analyzed by one-way ANOVA. **F**, **G** Short-term response after LPE application represses in particular growth and developmental-associated gene expression. Error bars show ± SD. Statistical significance was analyzed by one-way ANOVA. Also, see Fig. S1C. (H) MAP-kinase activation assay at 0, 0.5, 6, and 24 h after LPE application evaluates the activation of MPK3 and MPK6 compared to a mock-treated control. Three biological replicates were performed with similar results

Furthermore, we identified GO terms for genes that were significantly upregulated, these described gene functions in peptide metabolism, response to salt stress, oxidative stress aging, and bacterium at 24 h after LPE application (Fig. 2A, Table S2). Whereas, downregulated genes are classified into GO terms for developmental and metabolic process, gene expression and auxin response (Fig. 2B). Interestingly, cluster analysis revealed a major upregulation of defense-associated pathways at 6 h followed by a predominate downregulation at 24 h (Fig. 2C). We identified genes with a strong peak of upregulation at 6 h, whose encoded proteins catalyze rate-limiting steps in the JA-biosynthesis (*AOC1, AOC2, AOS, JOX4*) and JA-signaling pathway (*ORA59, PDF1.2, JAZ6, PR4, and WAX1*) (Fig. 2C, D, Fig. S1B). Genes that are predominately downregulated at 6 h and upregulated after 24 h following LPE administration contribute to small molecular metabolic and nucleotide processes, and response to light intensity and photosynthesis (Fig. 2F, G, Fig. S1C). Thus, it is conclusive that LPE amends the transcript profile predominately towards JA and ethylene coordinated immunity, while gene expression profiles that promote metabolic processes are neglected.

To analyze whether LPE perception triggers MAP kinase signaling pathways, we analyzed the phosphorylation of MPK3 and MPK6 which coordinate PTI and ETI in plant immunity (Meng & Zhang 2013). Previously, it was shown that the phospholipid, phosphatidic acid, binds to and triggers MPK3 and MPK6 phosphorylation in response to submergence-induced hypoxia (Zhou et al. 2022). This prompted us to analyze the phosphorylation of these immune MAP kinases at 0 h, 0.5 h, 6 h, and 24 h after LPE-treatment of 14-days-old seedlings (Fig. 2H). We could not detect changes in the phosphorylation status of MPK3 and MPK6 at the indicated time-points compared to mock-treated control which suggests that these immune-associated MAP kinase-signaling pathways play a minor role in the LPE-induced immunity.

### Altered *in-situ* LPE-homeostasis impacts plant resistance

We questioned whether changed *in-situ* LPE levels increase plant resistance according to exogenous LPE application. To address this question, we analyzed the mutants of *LYSOPHOSPHATIDYLETHANOLAMINE ACYLTRANSFERASE1* (*LPEAT1*) and *LPEAT2*. The *lpeat1* and *lpeat2* mutants were shown to have an increased *in-situ* homeostasis of LPE (Jasieniecka-Gazarkiewicz et al. 2017). We found that disease resistance of *lpeat1* and *lpeat2*-defective plants infected with *B. cinerea* was increased compared to the control group (Fig. 2E). Conversely, we analyzed the mutant of the PHOSPHOLIPASE 2-ALPHA (PLA2-ALPHA) compromised in LPE biosynthesis and quantity deficiency (Lee et al. 2010; Wang 2005). The *pla2*-*alpha* mutant exhibited an increased susceptibility to *B. cinerea* infection compared to WT and *lpeat1* and *lpeat2* (Fig. 2E). Thus, we found that increased *in-situ* LPE levels reduce the susceptibility of the *B. cinerea-*infected plants according to the exogenous application. However, the diminished *in-situ* LPE levels increase the susceptibility and vulnerability of the host. Taken together, the above results indicate that the modification of *in-situ* LPE abundance directly impacts plant resistance reminiscent of the exogenous administration.

### Hormonal signaling mutants show distinct susceptibility after LPE pretreatment

**Fig. 3:**
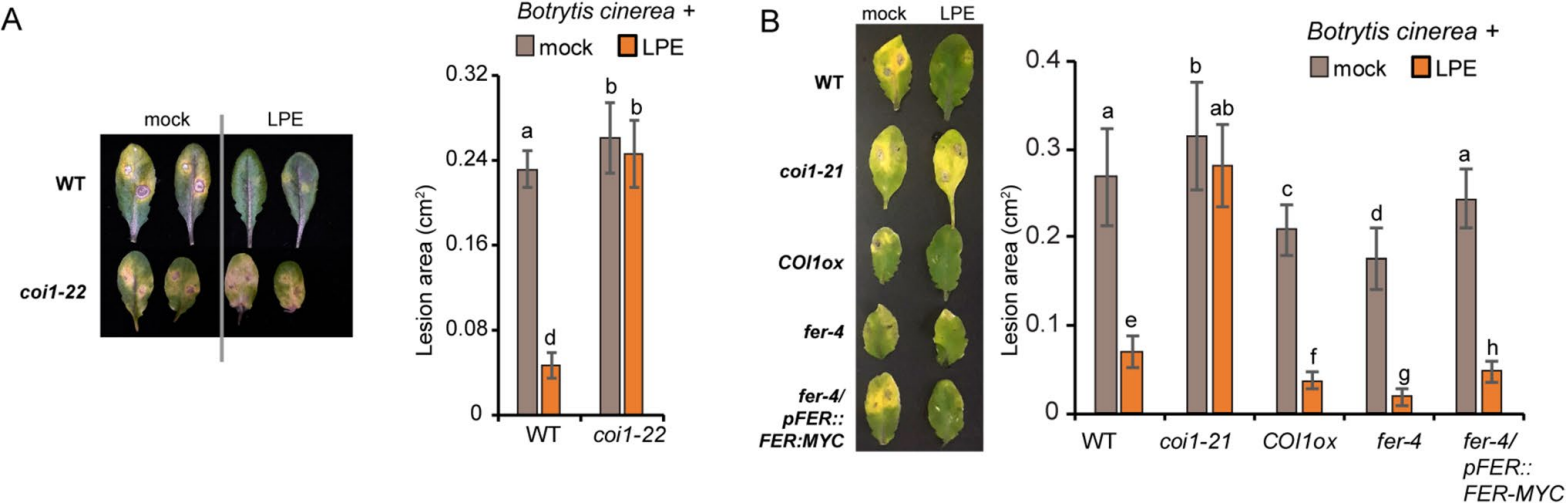
Jasmonic acid signaling mutants show a distinct susceptibility to *Botrytis cinerea* infection after LPE-pretreatment. **A**, **B** Infection experiments by the use of various hormonal mutants contributing to jasmonic acid signaling are depicted. Two allelic mutants of the JA-receptor COI1 (*coi1-21, coi1-22*), a *COI1*-overexpressor line (*COI1ox*), the JA-hypersensitive mutant *feronia* (*fer-4*) and the *feronia*-complementation line were analysed at 24 h after LPE-pretreatment and at 72 h after *Botrytis cinerea* infection. Error bars show ± SD. Three biological replicates were performed with similar results. Error bars show ± SD. Three biological replicates were performed with similar results. Statistical significance was analyzed by one-way ANOVA

**Fig. 4:**
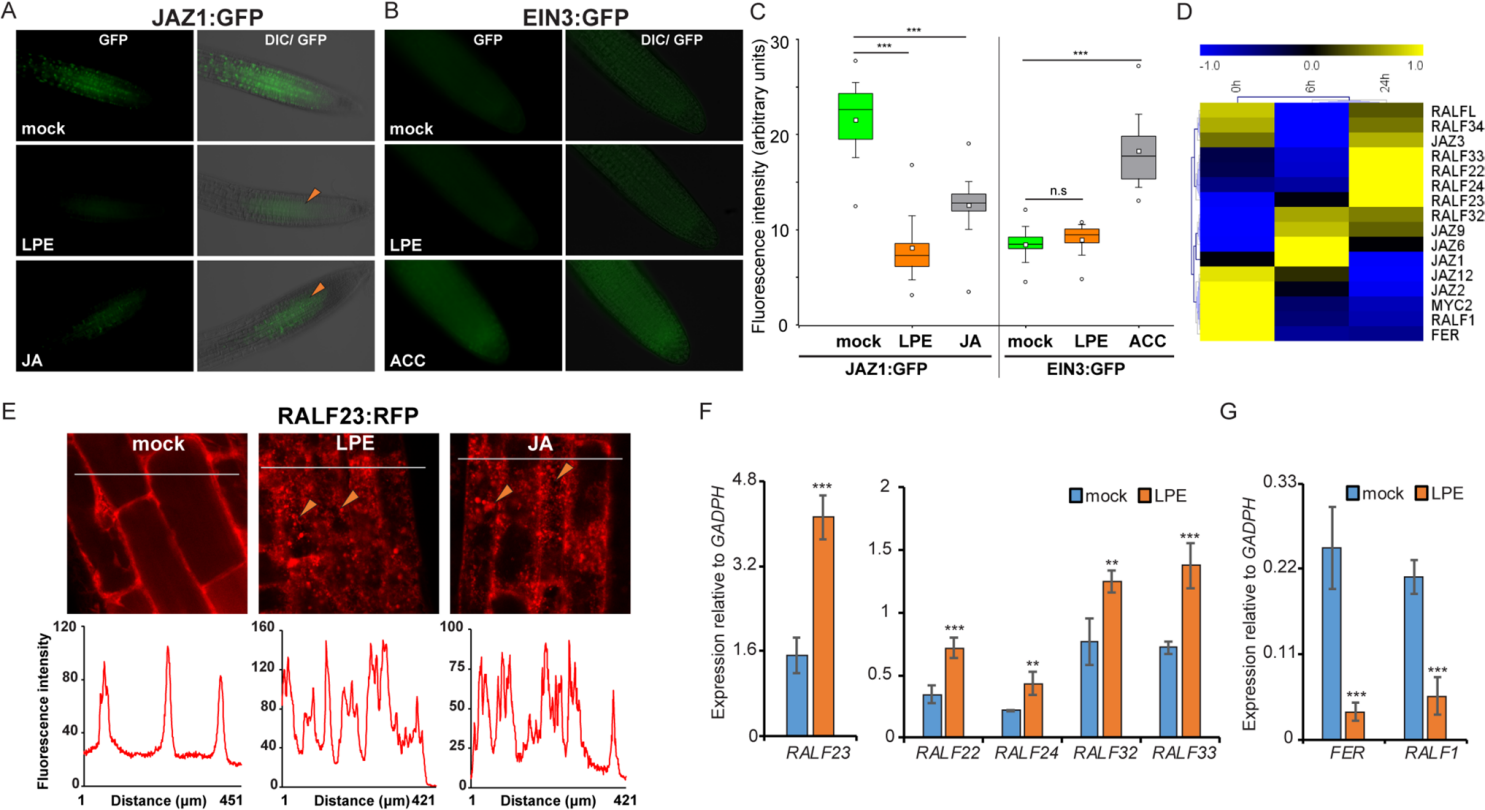
LPE targets the RALF23-FERONIA signaling module. **A**, **C** The JA-repressor JAZ1 linked to a GFP (JAZ1:GFP, yellow arrow) is degraded at 1 h after LPE-treatment and JA-application. Statistical significance was analyzed by one-way ANOVA **B**, **C** The protein stability of the key factor in the ethylene-signaling component EIN3 is not affected by LPE-application. By contrast, the ethylene precursor ACC triggers degradation of EIN3 at 1 h after administration. **D** Transcriptional profile of JA-signaling components and members of FERONIA-RALF signaling complex are differentially expressed at 0 h, 6 and 24 h after LPE-application. **E** Protein localization of RALF23-RFP at 1 h after LPE- and JA-application in root tips of stable transgenic *Arabidopsis* lines. RALF23-RFP internalizes in intracellular speckles (yellow arrow) after LPE-treatment. RALF23-RFP localizes in the plasma membrane under mock-treated conditions. Three biological replicates were performed with similar results. Thin gray bar within the images refer to the area used for the fluorescence intensity determination. **F**, **G** Quantitative PCR of the FERONIA (FER)-signaling repressors *RALF22, RALF23, RALF24, RALF32, RALF33* and of the FERONIA-signaling promotor *RALF1* and *FERONIA* at 24 h after LPE-treatment. Error bars show ± SD. Three biological replicates were performed with similar results. Statistical significance was analyzed by one-way ANOVA.

The transcript profile after LPE treatment revealed the enrichment of JA signaling genes which were previously shown to promote defense-response. Thus, we analyzed JA signaling mutants for changes in the immune-promoting effect given by LPE pretreatment and followed by *B. cinerea* infection. We characterized the two allelic mutants of the JA-receptor COI1 (*coi1-21, coi-22* (He et al. 2012)). Both allelic *coi1* mutants showed a higher susceptibility to *B. cinerea* after mock pretreatment compared to WT. After LPE pretreatment, a strong decrease in the *B. cinerea* infection could be observed in WT (Fig. 3A). However, both allelic *coi1* mutants showed a susceptibility to *B. cinerea* after LPE-pretreatment that corresponds to the *coi1* mock-treated control, indicating that JA-signaling is vital for the innate immunity-priming effect of LPE (Fig. 3A, B). To further analyze whether increased JA-signaling might promote the immunity-boosting effect of LPE, we included the *fer-4* mutant (Guo et al. 2018; Escobar-Restrepo et al. 2007), which exhibited an elevated JA-response in an MYC2-dependent manner. Furthermore, we analyzed the *COI1*-overexpressor line (*COI1ox*) driven by the *COI1* promoter in the WT plant. The mock-treated *fer-4* and *COI1ox* lines exhibited reduced susceptibility to *B. cinerea.* After LPE pretreatment, we intriguingly found that *COI1ox* and *fer-4* showed a further increased resistance to *B. cinerea* infection, when compared to LPE-pretreated WT plants (Fig. 3B). Together, these results indicate that the immune-promoting effect given by LPE pretreatment relies on JA-signaling and response. To analyze the impact of LPE on JA- and ET-signaling, we examined pivotal factors involved in these signaling pathways on their protein stability. Firstly, we analyzed the key transcription factors in the ET-signaling pathway ETHYLEN-INSENSITIVE 3 (EIN3). In the absence of ethylene, EIN3 is degraded by the 26S proteasome (Heydlauff et al. 2021; Binder 2020). In the presence of ET, EIN3 is stabilized and promotes ET signaling. We found the stabilization of EIN3:GFP (Volz et al. 2013) driven by the EIN3 promoter after the application of the ET-precursor ACC (Fig. 4B, C), Yet, after mock and LPE treatment we could not detect an EIN3 stabilization, which suggests that applied-LPE exerts minor effects on ET production and signaling. Secondly, we analyzed components of the JA-signaling pathway. The JA-signaling repressor JAZ1 is degraded at high JA levels and inhibits JA-signaling by targeting MYC2 at JA-default levels (Grunewald et al. 2009). We found JAZ1:GFP degradation after LPE and JA administration (Fig. 4A, C) in contrast to the mock treatment. This result suggests that LPE triggers the 26S proteasome-mediated degradation of JAZ1 thereby promoting JA-signaling.

### LPE targets the RALF23-FERONIA signaling module

The peptide hormones of the RALF group are involved in a multitude of biological signaling processes (Xiao et al. 2019), e.g., the inhibition of the FERONIA-mediated degradation of MYC2, thereby promoting JA-signaling and response (Guo et al. 2018).

Interestingly, we found a big subclass of *RALF*s (*RALF22, RALF23, RALF24, RALF32, RALF33, RALFL*) upregulated at 24 h after LPE application (Fig. 4D, F, G). RALF22 and RALF23 were shown to exert a pivotal function in the inhibition of the FER-mediated MYC2-degradation to favor JA-signaling. By contrast, FER and its interaction partner in RIPK-mediated RPM1-induced immunity, RALF1, are strongly downregulated after LPE administration (Fig. 4D, G). Likewise, several members of the MYC2-targeting JA-signaling inhibitors, *JAZ*s (e.g. *JAZ1*, *JAZ2*, *JAZ6*, *JAZ12*) show a reduced transcript amount after LPE treatment (Fig. 4D).

Studies have shown that RALF23 inhibits FER function thereby supporting MYC2-mediated JA-response (Guo et al. 2018). The FER-RALF module coordinates cell-wall sensing which in turn affects their subcellular localization in response to various stresses (Herger et al. 2019; Zhao et al. 2018). Thus, we analyzed the localization of RALF23 linked to RFP after LPE, JA, and mock treatment. We found that RALF23 internalizes in intracellular speckles at 1 h after LPE and JA treatment (Fig. 4E). This result suggests the involvement of the FER-RALF23 signaling module in LPE-responding molecular processes.

### Distinct ROS levels after LPE treatment in JA-signaling mutants

The elevation of *in-situ* reactive-oxygen species (ROS) homeostasis and the burst of ROS are among the first steps in the response to a pathogenic threat after the perception of pathogenic signatures (Nakagami et al. 2005). In this regard, the JA-mediated defense response is intimately coupled with changes in ROS homeostasis to oppose a necrotrophic invader (Stenzel et al. 2003). We previously showed that LPE increases the levels of *in-situ* hydrogen-peroxide H_2_O_2_ levels which are accompanied by the strong induction of H_2_O_2_ inducible gene expression (Volz et al. 2021).

**Fig. 5:**
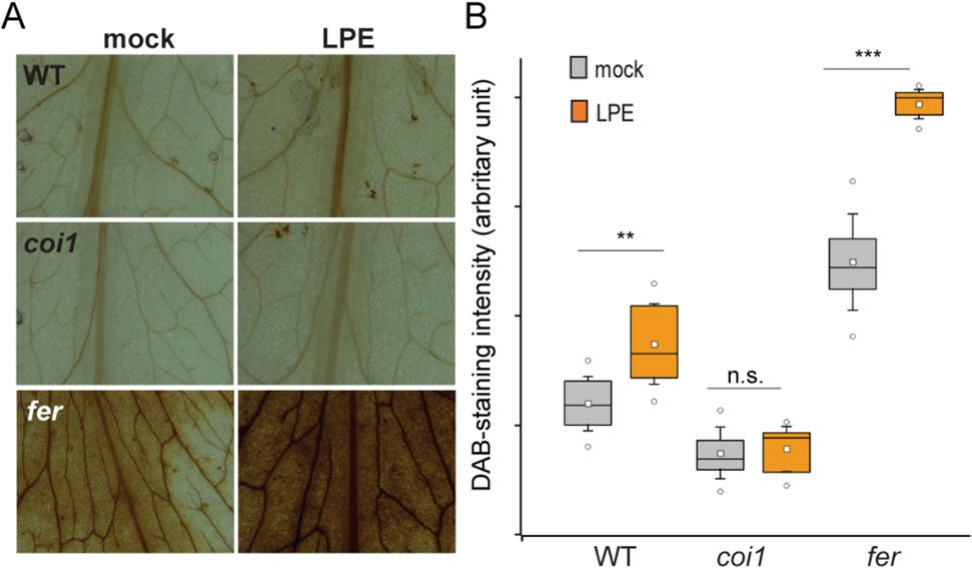
Reactive-oxygen species are vital for the immunity-promoting effect of LPE. **A**, **B** Distinct in situ hydrogen peroxide levels in WT, *coi1-21,* and *fer-4* following LPE application. Quantification of ROS-intensity units (arbitrary) was determined by ImageJ. Statistical significance was analyzed by one-way ANOVA

Here, we analyzed whether the distinct defense responses of WT, *coi1-21,* and *fer-4* following LPE pretreatment can be traced back to differences in their adaptive H_2_O_2_-homeostasis, by performing 3,3-diaminobenzidine (DAB) histochemical staining. At 24 h after LPE application, the ROS levels in WT and in particular in *fer-4* are significantly increased compared to the mock-treated counterpart (Fig. 5A, B). However, we could not detect changes in the ROS accumulation in *coi1* after LPE treatment thereby suggesting that the JA-signaling pathway contributes to the LPE-triggered changes in ROS homeostasis.

## Discussion

The use of bioactive molecules to modify the plant defense response is an important area of study. However, the role of cell membrane lipids in plant immunity is not well understood. We discovered that the cell membrane-localized phospholipid LPE promotes defense-associated gene expression and primes the plant immune system. The plant cell wall is one of the major carbon sources for necrotrophic pathogens. To penetrate these barriers and assimilate further nutrients from plant cells, pathogens devour their hosts by secreting cutinases and other cell wall degrading enzymes. Lipid priming is a process by which certain lipids, such as LPE, or lipid-derived molecules act as signaling molecules, preparing the plant's defense system to mount a faster and stronger response against potential threats, such as pathogen attacks. This phenomenon is an essential part of the plant’s immune response and is considered a critical aspect of plant defense. One of the well-known lipid signaling molecules involved in lipid priming is jasmonic acid, which is an oxylipin derived from the oxidation of fatty acids (Wasternack 2007; Wasternack & Strnad 2016). Oxylipins are produced when plants are subjected to various biotic (pathogens, insects) and abiotic stresses (mechanical damage, environmental factors). Oxylipins play a crucial role in signaling and regulating defense responses, including the activation of defense-related genes, synthesis of antimicrobial compounds, and the reinforcement of the plant cell wall to prevent pathogen ingress. Phytopathogens often have to overcome these lipid-mediated defense responses to establish successful infections. Researchers study the mechanisms behind lipid priming and how different lipids interact with the plant's defense signaling pathways to gain insights into developing more robust and sustainable methods for crop protection.

LPE application increases resistance to the necrotrophs *B. cinerea* and *C. heterostrophus* in Arabidopsis but this effect is eradicated in the *coi1* and amplified in the *fer-4* mutant. LPE promotes JA-signaling by degrading JAZ1, and ROS-homeostasis is necessary for LPE-mediated immunity. LPE might also repress FER-signaling by promoting the RALF function. Our findings suggest that LPE triggers immunity-associated signatures via affecting the FERONIA-COI1 mediated JA-signaling and ROS-homeostasis.

The concept of lipid priming has promising applications in agriculture. By priming crop plants with jasmonic acid or its analogs, farmers may improve crop resistance to pathogens and pests, reducing the reliance on chemical pesticides and promoting sustainable agriculture practices. Modifying *in-situ* LPE homeostasis in crops via the genomic engineering of LPE homeostasis might give rise to the improvement of crop stress resistance. To challenge this concept, we analyzed mutants that are affected by the biosynthesis and catabolism of LPE. The disruption of *LPEAT1* and LPEAT2 results in an increased *in-situ* level of LPE. Vice versa, we analyzed the *phospholipase 2alpha* mutant (*pla2alpha*) which is deficient in its level and production of LPE. After *B. cinerea* infection, we detected reduced lesion formation and increased resistance in *lpeat1* and *lpeat2*. By contrast, *pla2alpha* exhibited stronger lesion formation and reduced resistance. In summary, we revealed that increased *in-situ* LPE levels exert an immunity-promoting effect in accordance with the exogenous LPE application. Importantly, these studies suggest that the genomic engineering of LPE levels in crops (*e.g.* rice) might be advisable for further research. We anticipate that crops with elevated LPE-homeostasis might be tolerant to facing biotic threats and abiotic stresses in a hostile environment. Thus, it might be advisable to generate rice lines, deficient in *LPEAT1* and *LPEAT2* with increased *in-situ* LPE levels, and to study their resistance to biotic threats, such as *B. cinerea*, *Magnaporthe oryzae,* and *C. heterostrophus*.

## Materials and methods

### Plant material and growth conditions

The *A. thaliana* ecotypes Columbia (**Col-0**) and Wassilewskija (Ws-0) used in this study were obtained from the *Arabidopsis* Biological Resource Center (ABRC, Ohio State University). In addition to these ecotypes, we used the following mutants and marker lines derived from Col-0:*, coi1-21* N68754*, coi11-22, fer-4 N69044, lpeat1* GK-825D08*, lpeat2* SALK_107699C*, pla2-aplha* SALK_099415C*, COI1ox* N2105631*,. p35S::JAZ1:GFP* N799800 were ordered by NASC.

*Arabidopsis thaliana* plants were grown on a mixture of commercial potting soil and perlite (3:1) or on Murashige and Skoog (MS) agar medium) in a growth chamber with 16/8 h day/night photoperiod, 22 °C and 80% relative humidity.

### LPE-pretreatment followed by infection experiments

Four- to five-week-old A. thaliana plants, which were grown in soil and irrigated from the bottom, were treated by spraying them with either a mock solution of 250 ppm Tween 80 or a solution of 50 ppm LPE (50 mg/l) in 250 ppm Tween 80, 24 h prior to inoculation with the pathogen. The plants were then placed under a hood and maintained at a temperature of 22 °C in the growth chamber.

*Botrytis cinerea* spores were prepared for each experiment by using a spore stock stored at − 80 °C in 25% glycerol. The spores were spread evenly on a PDA medium and incubated at 24 °C for 14 days. After harvesting, the spore concentration was determined using a hemocytometer. Leaves were inoculated with a 4 µl droplet of a spore suspension (5 × 10^5^ spores/ml) and analyzed after 72 h post-infection (hpi). At least 30 plants of each plant genotype were sampled in three biological replicates.

*Cochliobolus. heterostrophus (*C4—*Tox1*^+^;*MAT-2*) conidia were grown on sucrose-proline agar at 22℃. Inoculation of 4-week-old A. thaliana plants with *C. heterostrophus* involved harvesting conidia from fungal cultures on sucrose-proline agar, and adjusting their concentration to 5 × 10^5^ conidia/ml with water. A total of 20 ml of the conidial suspension was sprayed on five plants using an air paintbrush connected to a compressor. The inoculated plants were incubated in a dew chamber for 16 h at 25 °C under 100% relative humidity and then transferred to a growth chamber at 22 °C and 80% relative humidity to observe disease development. Each inoculation experiment was repeated three times. To assess disease severity, a numerical scoring system based on the percentage of diseased leaf area (DLA) was used. ImageJ software was used to determine DLA based on brightness intensity values at 3 and 6 days post-inoculation (dpi). DLA scores ranged from 0 to 5, with 0 indicating no necrotic or chlorotic flecks on the leaves (water controls always received a score of 0). The numerical values corresponded to the percentage of leaf area showing necrosis or chlorosis, with 1 representing 1–20%, 2 representing 21–40%, 3 representing 41–60%, 4 representing 61–80%, and 5 representing 81–100%.

### Immunoblot

Nuclear proteins were extracted from 14-day-old seedlings grown on half MS-medium. The proteins were quantified using the Bradford method, and equal amounts of proteins were separated by SDS-PAGE. The separated proteins were then transferred to a polyvinylidene difluoride membrane (Bio-Rad) using a Mini-Protean 3 Cell (Bio-Rad). Immunoblot analysis was carried out using primary polyclonal pTpY antibodies at a concentration of 1 µg/mL, followed by secondary antibodies conjugated to alkaline phosphatase. Chemiluminescence was used to detect the antibody complexes using the Immun-Start AP Substrate kit (Bio-Rad). The experiment was repeated three times, and a representative result from one bioRep is shown.

### Quantitation of immunoblot membranes

To measure protein levels in extracts and guarantee uniform loading of all proteins for gels used in immunoblot analysis, Bradford assays were utilized.

### Molecular cloning

The coding sequence of *RALF23* was amplified form an Arabidopsis seedling cDNA pool without an STOP codon and cloned into the Gateway-based *pENTR-D-Topo* vector. After sequencing the *RALF23* cds, it was cloned into the *pDEST::C-RFP-Stop* plasmid to generate *pDEST::RALF23:RFP*. Subsequently, *pDEST::RALF23:RFP* was used to transform Arabidopsis Col plants by employing the Agrobacterium-mediated transformation.

### Histochemical staining and ROS-burst assay

Histochemical staining using 3,3′-diaminobenzidine (DAB) was used to perform in situ detection of H_2_O_2_ in 4-week-old A. thaliana plants treated with LPE (50 mg/l) or mock solution, following a previously described protocol. In brief, leaves were incubated in DAB solution (50 mg DAB, 130 mg Na2HPO4, 0.01%v/v Tween 20) for 6 h. Subsequently, the leaves were mounted in 20% glycerol. This experiment was repeated three times, and a representative result is shown.

### RNA-extraction, cDNA synthesis and qPCR

To analyze the expression levels of individual marker genes, RT-PCR was conducted on plants grown on plates containing half-strength Murashige and Skoog (MS), 0.5% sucrose, 1% agar, and 0.5% MES at pH 5.7. Total RNA was extracted from LPE (50 mg/l) and mock-treated 14-day-old seedlings using the easy-spinTM Total RNA Extraction Kit. Reverse transcription was performed using SuperScript II reverse transcriptase, and RT-qPCR was carried out using an Applied Biosystems 7500 Real-Time PCR systemTM and an SYBR green PCR master mix. Data were averaged from duplicates of at least three biological replicates, and oligo-nucleotides used to determine transcript levels.

**Table:** 

RV4s	GAATCGGTCGTTTGGTTGCTA	*qPCR GADPH*
RV4as	TTAACAGCGACGAGCTCAACAT	*qPCR GADPH*
RV15s	GTACCGCCGTCTAAGGACAT	*qPCR AOC2*
RV15as	CACAGCGATACGAGAAACATT	*qPCR AOC2*
92 s	CGTTGTTCAAGCACCAGAGA	*qPCR MEE14, CBP1*
92as	CGGATTCGGCTTTTTCAATA	*qPCR MEE14, CBP1*
93 s	CGTACGTGAATGGCAAGCTA	*qPCR NAD(P) BP AT2G29170*
93as	GGCAACGTTTTCCATGAGTT	*qPCR NAD(P) BP AT2G29170*
94 s	CGTGGCTCGTGTGATAAAGA	*qPCR SAG29*
94as	CGGCGCTTATAGTGAGGAAG	*qPCR SAG29*
95 s	TGCTTACGTTGGACAGCTTG	*qPCR NPF6*
95as	ACTCACGAAGAATCCCATCG	*qPCR NPF6.4*
96 s	CGGCTTTTGCTTAGTTCCTG	*qPCR COL7 CONSTANS-LIKE 7*
96as	CCATGATTGCCTTCTCGACT	*qPCR COL7 CONSTANS-LIKE 7*
165 s	TCGCCGTATCTTCTCAATCC	*qPCR RALF23_*
165as	CAGAGCACTCGGCAATTGTA	*qPCR RALF23_*
166 s	TCTCCGTCACAGTTCCGATT	*qPCR RALF24_*
166as	ATCTCCGATGGCATCATCTC	*qPCR RALF24_*
167 s	AACAGTGTGCCTTGTTCACG	*qPCR_RALF22_*
167as	CTGTAAGGATTCGCCTGAGC	*qPCR_RALF22_*
168 s	TCTTGGTCAAGCCAGAGGTT	*qPCR_RALF32_*
168as	AAGACTCGCCTCGTTTACCA	*qPCR_RALF32_*
169 s	ACTCTCCACAAAACCCGTTG	*qPCR_RALF33_*
169as	CGACTCGATCGGTACGAAAT	*qPCR_RALF33_*
173 s	GGCGTTGTTCTATTCGAAGC	*qPCR Feronia*
173as	TGTTCCTTTGCAAGTGTTGG	*qPCR Feronia*
174 s	CGACTGGAGACAATGGTTCA	*qPCR RALF1*
174as	TTGTGGTCGCCAATATTCTTC	*qPCR RALF1*
182	ACGGTCTCAACGCTACCAAC	*fer-4* genotyping
183	TTTCCCGCCTTCGGTTTA	T-DNA of fer4
184 s	GATTACTCTCCAACAGAGAAAATCCT	*fer-4* genotyping
184as	CGTATTGCTTTTCGATTTCCTA	*fer-4* genotyping
185 s	CTG TAA GCA GTT GAA GCG GCT GAG GAT TGA A	coi1-22
185as	GTC TCA GAT AGA ATG CAA ATC GTC TGA GTT TCT TGG AT	coi1-22 BamH1
186 s	GAC AAC ACT TGT TGT TTT TCT TCA GAC AAG GAA TGT AAC CG	coi1-21
186as	GGT CGA GTA AGA CAA GGC GGA AGT CAC AGA GGT T	coi1-21 HpaII
232as	TTATCACCGACCATTGTTGCATCG	*lpeat2* mutant, SALK_107699C
233as	TGCTCCAACTATTATGCTTTTTCCAG	*lpeat1* mutant, GABI_825D08
234	CCATATTGACCATCATACTCATTGC	Left-Border for T-DNA (Gabi-Kat; pAC161)
235	CAAGTGGATTGATGTGATATCTCC	Right-Border for T-DNA (Gabi-Kat; pAC161)
236 s	AAAGTCATGTAGCCTAACACGTC	*lpeat2* mutant, bind on 9.exon, with 232as
237 s	TTTGACTAAGATTACCATTGAGAG	*lpeat1* mutant, bind on 9.exon, with 233as
238 s	tcgatatacagaagttctttgagc	T-DNA line for *ATSPLA2-ALPHA*, AT2G06925
238as	ttagccatacgaaacaaatgagtc	T-DNA line for *ATSPLA2-ALPHA*, AT2G06925

### RNA-sequencing and bioinformatics analysis

The RNA sequencing was performed using Illumina sequencer (NICEM, Seoul, Korea). The quality of the raw sequence reads was checked using FastQC v0.11.9 (Andrews 2017), and the adaptors and poor reads were removed by Fastp v0.20.1 (Chen et al. 2018). The quality-checked reads were mapped to the reference genome TAIR10 using HISAT2 v2.1.0 aligner (Kim et al. 2019). The mapped reads were sorted using SAMsort in Picard v2.23.3 (Broad Institute). Read counting was performed using StringTie v2.1.3 (Pertea et al. 2015), and differentially expressed gene analysis was executed by DESeq2 (Love et al. 2014). The gene ontology enrichment test was performed with TAIR10 data in AgriGO v2.0 (Tian et al. 2017). Three biological replicates for mock, 6 h and 24 h were analyzed.

### Statistical analysis

Statistical significance was determined using one-way ANOVA with Tukey post-test. The significance level was set at p ≤ 0.05, and different letters above the bars indicate significant differences. Samples sharing letters are not significantly different. Asterisks were used to indicate the level of significance, with n.s. representing non-significant results, *indicating *p* ≤ 0.05, **p ≤ 0.01, and ***p ≤ 0.001.

### Supplementary Information

Below is the link to the electronic supplementary material.

Supplementary file1 (TIF 25517 KB) Fig S1: Differentially-regulated genes after LPE-application. (A) Number of up and downregulated genes in the LPE-transcriptome study. (B) Upregulated defense-associated pathways at 6 hrs followed by a predominate downregulation at 24 hrs. (C) Differentially-regulated genes contribute to the JA-biosynthesis (AOC1, AOC2, AOS, JOX4) and JA-signaling pathway (ORA59, PDF1.2, JAZ6, PR4, and WAX1) Predominately downregulated at 6 hrs and upregulated after 24 hrs following LPE application are associated to small molecular metabolic and nucleotide processes, and response to light intensity and photosynthesis. (D) Expression study of PDF1.2 and ORA59 6 hours after mock and LPE-application.

Supplementary file2 (XLSX 450 KB) Table S1: Differentially regulated genes (up and downregulated) 6 hours after LPE application, p<0.01

Supplementary file3 (XLSX 677 KB) Table S2: Differentially regulated genes (up and downregulated) 24 hours after LPE application, p<0.01

## Data Availability

All relevant data can be found within the manuscript and in its Supporting Information online at the publisher’s website.
