# Supplementary figures and images for: Lyso-phosphatidylethanolamine triggers immunity against necrotrophs by promoting JA-signaling and ROS-homeostasis
Source: Plant Mol Biol. 2023 Dec 12;113(4-5):237–47. doi: 10.1007/s11103-023-01385-x (PMC10721665; doi:10.1007/s11103-023-01385-x)

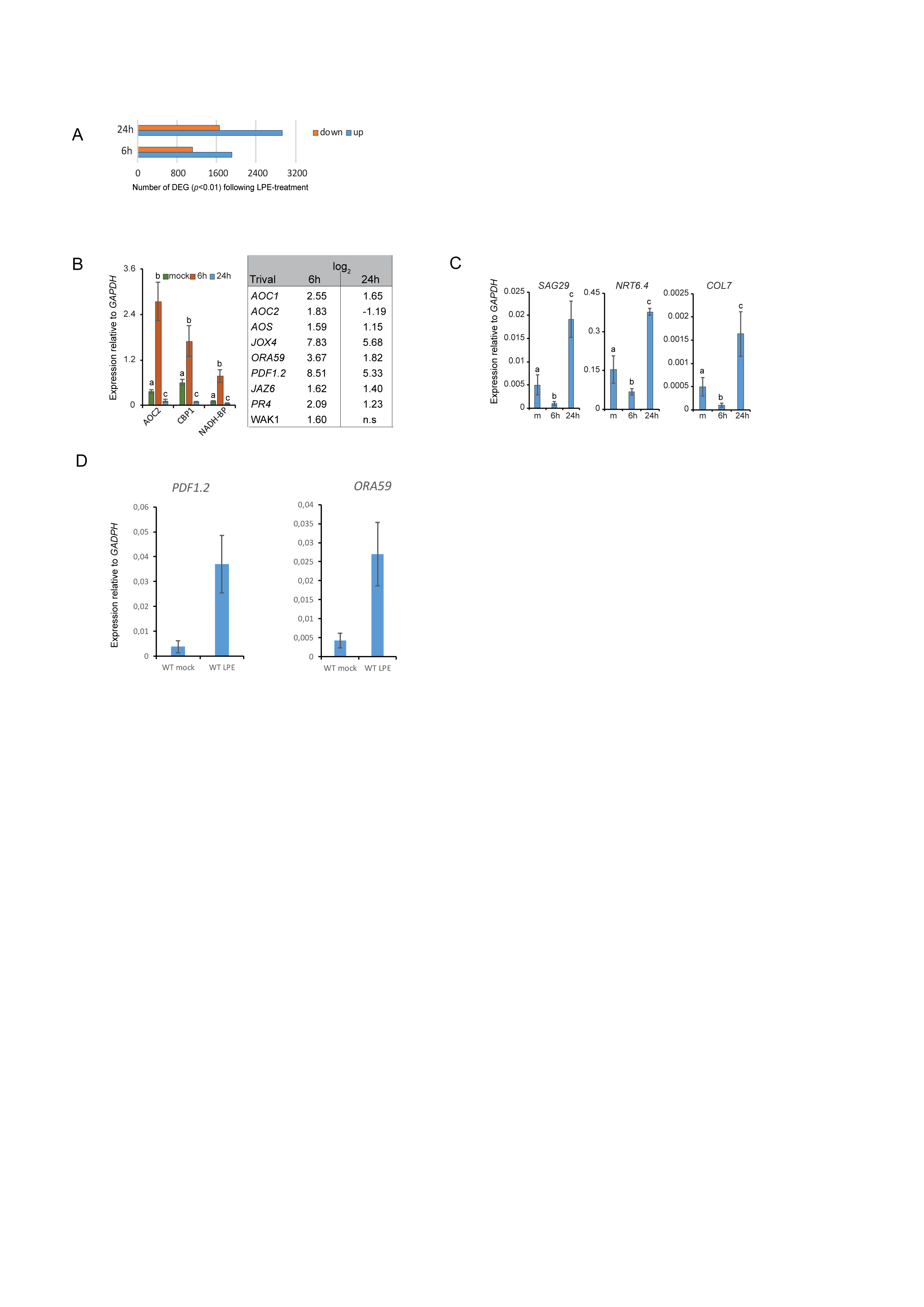

Supplement: Supplementary file 1 — Supplementary file1 (TIF 25517 KB) Fig S1: Differentially-regulated genes after LPE-application. (A) Number of up and downregulated genes in the LPE-transcriptome study. (B) Upregulated defense-associated pathways at 6 hrs followed by a predominate downregulation at 24 hrs. (C) Differentially-regulated genes contribute to the JA-biosynthesis (AOC1, AOC2, AOS, JOX4) and JA-signaling pathway (ORA59, PDF1.2, JAZ6, PR4, and WAX1) Predominately downregulated at 6 hrs and upregulated after 24 hrs following LPE application are associated to small molecular metabolic and nucleotide processes, and response to light intensity and photosynthesis. (D) Expression study of PDF1.2 and ORA59 6 hours after mock and LPE-application. [file 11103_2023_1385_MOESM1_ESM.tif]
